# The Effect of Altering the Mechanical Loading Environment on the Expression of Bone Regenerating Molecules in Cases of Distraction Osteogenesis

**DOI:** 10.3389/fendo.2014.00214

**Published:** 2014-12-10

**Authors:** Mohammad M. Alzahrani, Emad A. Anam, Asim M. Makhdom, Isabelle Villemure, Reggie Charles Hamdy

**Affiliations:** ^1^Division of Orthopaedic Surgery, Shriners Hospital for Children, Montreal Children Hospital, McGill University, Montreal, QC, Canada; ^2^Department of Orthopaedic Surgery, University of Dammam, Dammam, Saudi Arabia; ^3^Department of Orthopaedic Surgery, King Abdulaziz University, Jeddah, Saudi Arabia; ^4^Department of Mechanical Engineering, École Polytechnique de Montreal, Montreal, QC, Canada; ^5^Sainte-Justine University Hospital Center, Montreal, QC, Canada

**Keywords:** mechanical loading, growth factor, distraction osteogensis, bone regeneration, bone regenerating molecule

## Abstract

Distraction osteogenesis (DO) is a surgical technique where gradual and controlled separation of two bony fragments following an osteotomy leads to the induction of new bone formation in the distracted gap. DO is used for limb lengthening, correction of bony deformities, and the replacement of bone loss secondary to infection, trauma, and tumors. Although DO gives satisfactory results in most cases, one major drawback of this technique is the prolonged period of time the external fixator has to be kept on until the newly formed bone consolidates thus leading to numerous complications. Numerous attempts at accelerating bone formation during DO have been reported. One specific approach is manipulation of the mechanical environment during DO by applying changes in the standard protocol of distraction. Attempts at changing this mechanical environment led to mixed results. Increasing the rate or applying acute distraction, led to poor bone formation in the distracted zone. On the other hand, the addition of compressive forces (such as weight bearing, alternating distraction with compression or by over-lengthening, and then shortening) has been reported to increase bone formation. It still remains unclear why these alterations may lead to changes in bone formation. While the cellular and molecular changes occurring during the standard DO protocol, specifically increased expression of transforming growth factor-β1, platelet-derived growth factor, insulin-like growth factor, basic fibroblast growth factor, vascular endothelial growth factor, and bone morphogenic proteins have been extensively investigated, the literature is sparse on the changes occurring when this protocol is altered. It is the purpose of this article to review the pertinent literature on the changes in the expression of various proteins and molecules as a result of changes in the mechanical loading technique in DO and try to define potential future research directions.

## Introduction

**Figure 1:**
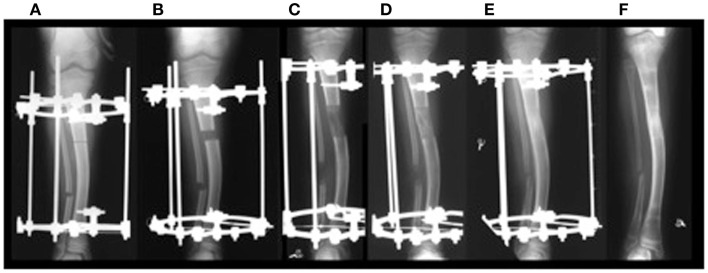
**(A)** Application of distractor; **(B)** start of distraction; **(C)** end of distraction; **(D,E)** consolidation phase without any distraction until bone in the distraction gap consolidates; **(F)** removal of distractor. ©2012 Hamdy et al. (7).

**Figure 2:**
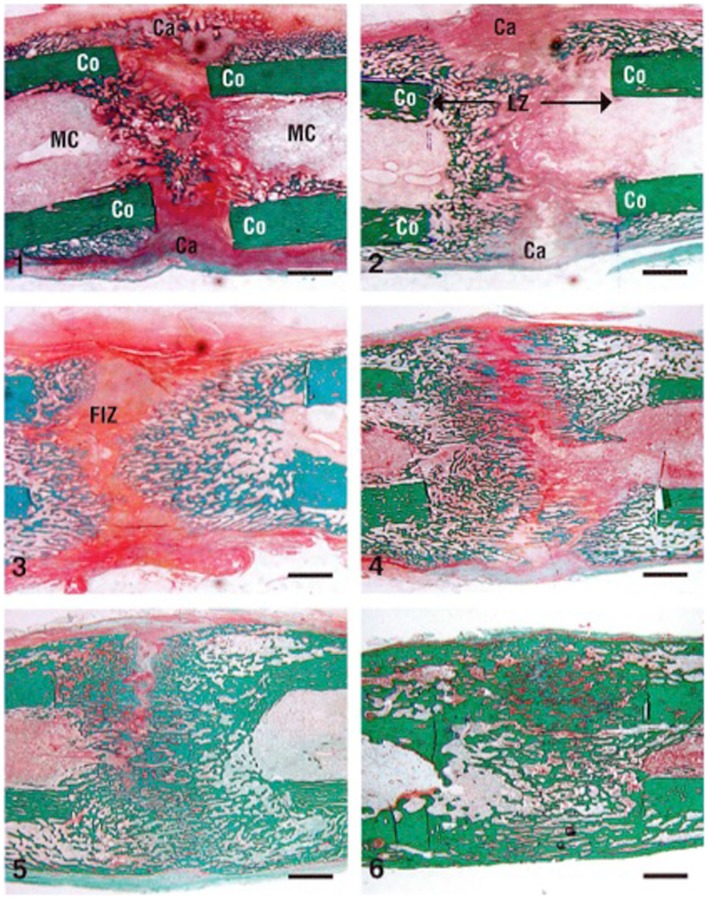
**Cellular changes in a rabbit DO model during distraction osteogenesis of the tibia (stain is Trichrome staining)**. The numbers indicate the number of weeks after the distraction process was started (1–3 are during the distraction phase and 4–6 are during the consolidation phase). Co, cortex; LZ, lengthened zone; Ca, callus; FIZ, fibrous interzone. Bar scale = 2 mm. Reprinted from Rauch et al. (34), with permission from Elsevier.

Distraction osteogenesis (DO) is a surgical technique first described by the Russian physician Ilizarov in the early 1950s (1, 2). This technique consists of performing an osteotomy to a bone that needs to be lengthened followed by gradual and controlled distraction of the two ends of the osteotomized bone. These mechanical forces of distraction lead to the induction and formation of new bone in the distracted gap (Figures 1 and 2) (1, 2). When the desired amount of lengthening is reached, the distraction is stopped but the external fixator is kept on until the newly formed bone in the distracted gap consolidates and becomes strong enough to withstand external forces after removal of the external fixator without bending or fracturing. The surgical technique of DO involves several temporal phases outlined below (3).

### Latency phase

The latency phase starts immediately following the osteotomy and lasts between 5 and 7 days. It allows the formation and organization of the hematoma and facilitates the recruitment of inflammatory cells and mesenchymal stem cells (4). This stage resembles the acute stage of fracture healing, including hematoma formation, immediate inflammatory response, and subsequent differentiation of stem cells into chondrocytes and osteoblasts (4).

### Distraction phase

In this phase, following the latency period, distraction of the two bone segments is started at a specific rate and rhythm of 1.0 mm a day, divided into four increments. This protocol was shown – experimentally and clinically – by Ilizarov to be the optimal rate and rhythm of distraction for bone formation. Higher rates of distraction lead to poor or delayed regenerate bone formation while slower rates of distraction lead to premature consolidation (3). This phase is characterized by the formation of a radiolucent central fibrous interzone (FIZ) in the middle of the distracted gap (Figure 2). The fibroblast cells and collagen fibers are arranged longitudinally along the axis of distraction. In addition, one of the hallmarks of this phase is formation of new blood vessels with intense angiogenesis, neoangiogenesis, recruitment of osteoblasts, and new bone formation (5).

### Consolidation phase

Once the desired amount of lengthening is obtained, distraction ceases, and the newly formed bone gradually bridges the gap between the two ends of the osteotomy (Figure 2). This new regenerate bone arises from the periosteum (hence, the importance of avoiding damage to the periosteum), the medullary canal, and the surrounding soft tissues (6). This phase is the longest phase in DO, about 1 month for each centimeter lengthened (1). In addition, it lasts until the newly formed bone in the distracted gap becomes biomechanically strong enough to allow removal of the fixator.

## Advantages of DO Over Other Techniques of Bone Regeneration

Distraction osteogenesis is widely considered the best *in vivo* tissue engineering and has numerous advantages over other bone graft techniques, such as autografts, allografts, vascularized fibular grafts, and various artificial bone substitutes (7). With the technique of DO, very large and almost unlimited amounts of new bone can be generated and this newly formed bone is vascularized and of the same micro and macrostructure as the native bone. Furthermore, DO leads to the generation of new bone in a reproducible and predictable manner. One other major advantage of DO is the simultaneous regeneration and lengthening of all soft tissues surrounding the lengthened bone, including skin, subcutaneous tissues, blood vessels, nerves, and muscles (8).

## Clinical Applications of DO

**Figure 3:**
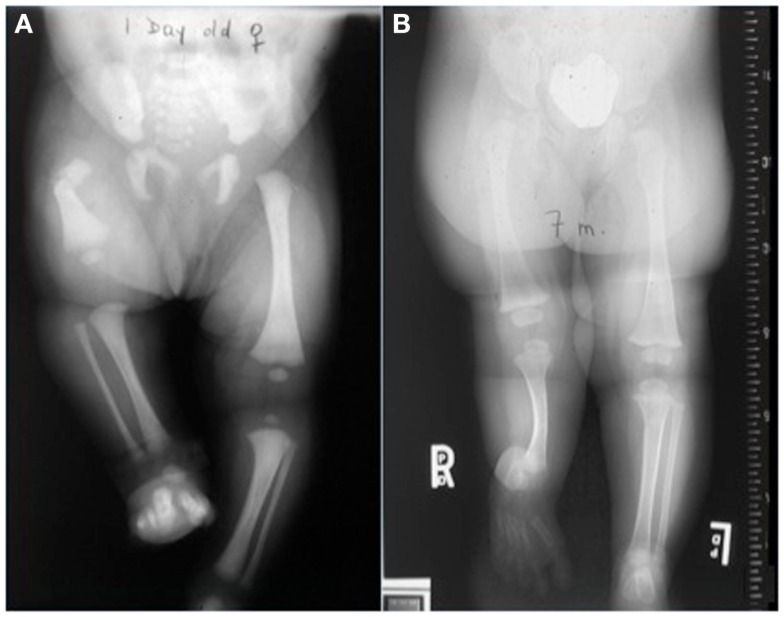
**Distraction osteogenesis is used to manage multiple orthopedic conditions including congenital short femur (A) and fibular hemimelia (B)**.

Nowadays, the technique of DO is widely used worldwide in the management of numerous orthopedic conditions including gradual correction of bony deformities, limb lengthening, and management of bone loss secondary to infection, trauma, and tumors (Figure 3) (7, 9). Currently, this technique has been applied in maxillofacial surgery for mandibular lengthening and in the treatment of craniofacial deformities (10). DO has also gained popularity in the field of dental surgery (11–13).

## Problems Associated with DO

**Figure 4:**
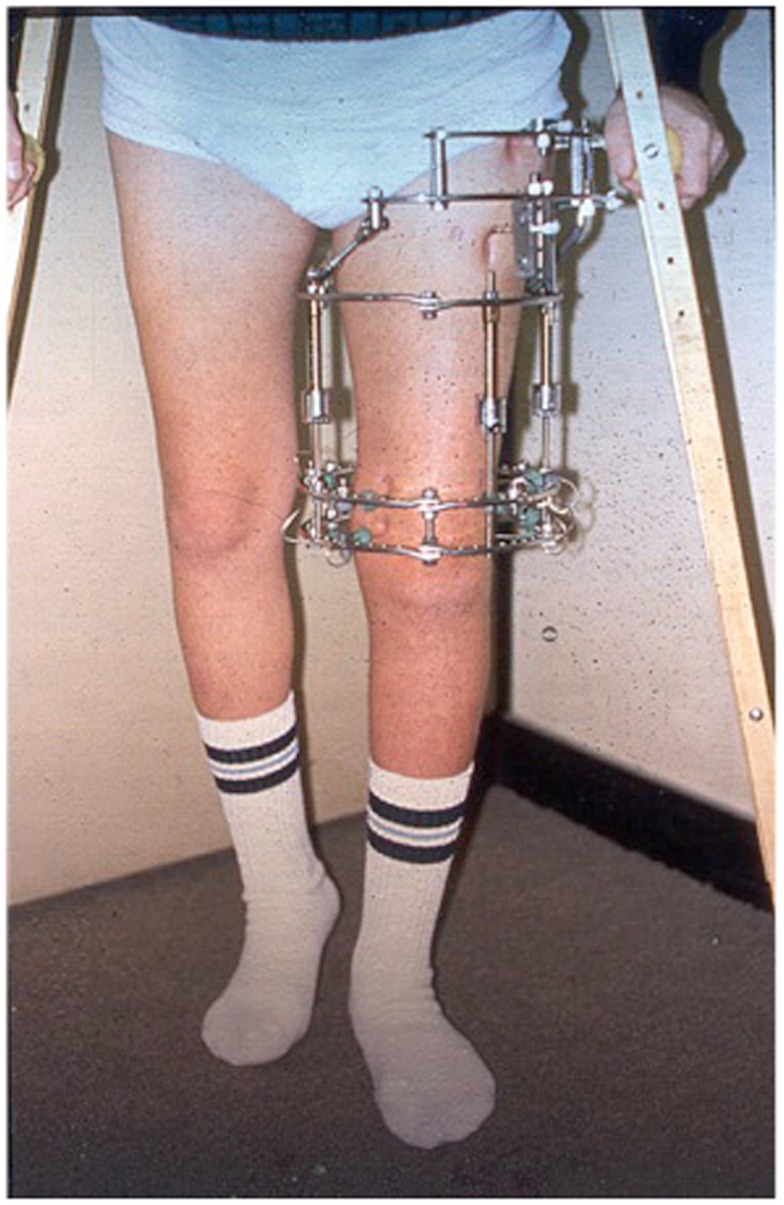
**Ilizarov ring fixator frame applied for distraction osteogenensis of the femur**.

Although DO gives satisfactory results in most cases, one of the drawbacks of this technique is the prolonged length of time the external fixator has to be kept in place until the newly formed bone in the distracted gap consolidates. For every centimeter lengthened, the fixator has to be kept in place for about a month. For example, a child undergoing a 6.0 cm lengthening will require the fixator to be kept in place for about 6 months. This prolonged length of time during which the fixator is kept in place, may increase the risk of complications, such as pin site infections, pain, discomfort, and psychological complications (Figure 4) (14, 15).

Numerous methods have been described in an attempt to accelerate the consolidation of the newly formed bone and hence allow early removal of the fixator (16). Most of these techniques are invasive and involve the local application of substances such as osteogenic growth factors, allografts, autografts, mesenchymal stem cells, various synthetic bone substitutes (such as TCP – tricalcium phosphates), the systemic application of bone stimulating pharmacological agents or the use of external stimulation such as electromagnetic fields (17).

**Figure 5:**
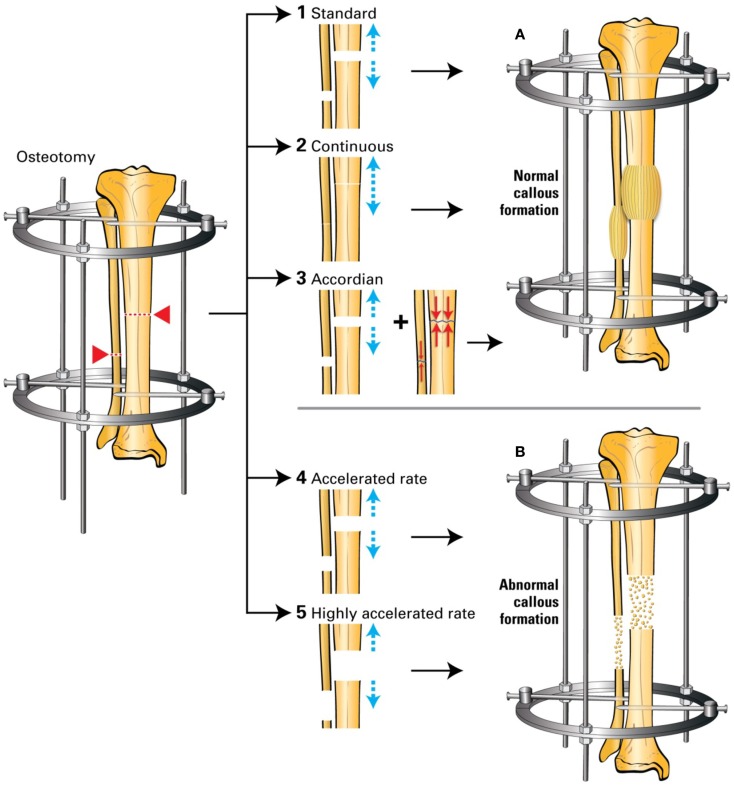
**(1) Standard DO: 0.25 mm/6 h to achieve 1 mm/24 h; (2) continuous DO: 0.02 mm/24 min to achieve 1 mm/24 h; (3) accordion maneuver: addition of compression to the standard distraction; (4) accelerated DO: 1.5 mm/24 h; (5) highly accelerated DO: 3 mm/24 h**. **(A)** Normal callus formation; **(B)** abnormal callus formation. Based on Ref. (24–26).

One area that has been surprisingly less extensively investigated in this context is the mechanical loading environment in DO and whether changes in this mechanical environment during the process of DO may have an impact on bone formation and consolidation (Figure 5) (18–20). Ilizarov discovered that when gradual and controlled distraction is applied to the two ends of an osteotomized bone, new bone would form in the distraction gap (1, 6). He called this phenomenon the “Law of Tension Stress.” In fact, without knowing it and long before the term mechanotransduction was coined for the first time by Harold Frost in the 1960s (21), Ilizarov laid the foundation of the whole field of mechanotransduction, where the mechanical forces of distraction applied during the process of DO are translated into molecular signals that lead to the induction of new regenerate bone (the tension of the distraction forces causes stress in the distracted tissue). This controlled distraction protocol described by Ilizarov more than 60 years ago consists of a rate and rhythm of distraction of 0.25 mm four times a day and does not include any compression forces besides those of distraction. This unique type of mechanical loading in the context of bone regeneration, revolutionized the field of bone regeneration, and continues to be followed almost to the letter worldwide in both long bone DO (9, 22) and mandibular DO (23).

It is surprising that in the present time and knowing the beneficial and anabolic effects of compressive forces and loading on skeletal tissue, this protocol remains largely unchallenged (2). In an attempt to accelerate bone formation in DO, several authors have analyzed the effects of applying various changes in the standard protocol by changing the rate or rhythm of distraction or by the addition of compressive forces that alternate with the distraction cycles (accordion technique). Some of these attempts – specifically, the addition of compressive forces – were successful in accelerating bone formation while others (increasing the rate of distraction) were not.

Although bone formation using standard protocol in DO has been extensively investigated at both the cellular and molecular levels, there have been very few reports analyzing the changes in the molecular expression of various proteins and molecules secondary to changes in the mechanical environment. It is the aim of this study to review the pertinent literature on that topic, try to identify potential therapeutic targets for accelerating bone formation and define future research directions.

## Molecular Changes during Standard Distraction Rate and Rhythm

The expression of various proteins and molecules and signaling pathways during the process of DO using the standard protocol – 1.0 mm distraction a day divided into four equal increments – has been extensively investigated in both human beings and animal models of DO (4, 27, 28). Compared to simple osteotomy without distraction, systemic up-regulation of transforming growth factor-β1 (TGF-β1), platelet-derived growth factor (PDGF), insulin-like growth factor (IGF), basic fibroblast growth factor (bFGF), vascular endothelial growth factor (VEGF), and its receptors VEGFR 1 and 2 have been reported by multiple investigators suggesting that these changes are caused by the distraction process (29–31).

**Figure 6:**
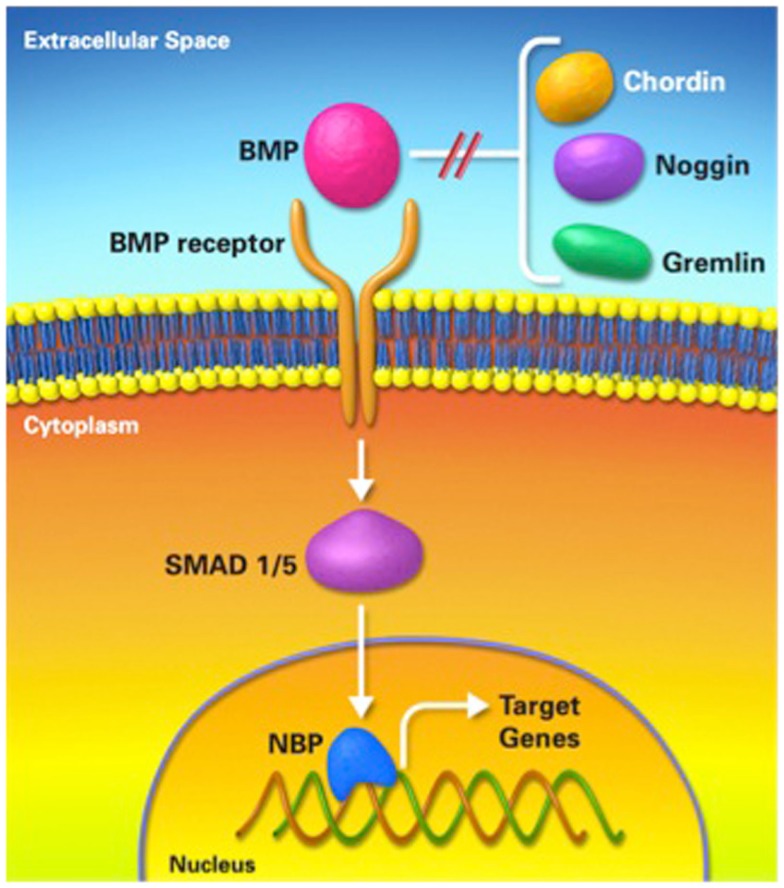
**Bone morphogenic protein pathway**. BMP; bone morphogenic protein, NBP; nuclear binding protein.

Using a standard DO protocol in various animal models (mice, rats, rabbits, dogs, and sheep), we and others have shown that the expression of numerous factors related to osteogenesis and chondrogenesis is mostly upregulated during the distraction phase, when the mechanical forces of distraction are applied and then, the expression of these factors is downregulated once the mechanical forces of distraction cease at the end of the distraction phase. These proteins include bone morphogenic proteins (BMPs); an extensively studied protein in the context of DO (Figure 6), in addition to TGF-β1, FGF, IGF, and PDGF (4, 28, 32–36).

In addition, the expression of extracellular matrix proteins collagen type 1, 2, 4, and 10, osteocalcin, osteopontin, and osteonectin during the various phases of DO has been reported in the literature, and showed highest expression during the distraction phase of this process and decreased expression toward the end of the lengthening process (33, 37–40).

Angiogenesis and neoangiogenesis factors have also been identified in the distraction zone during DO, specifically members of the VEGF and angiopontin signaling pathways (5, 41–45).

Pro-inflammatory cytokines involved in bone repair [interleukin-6 (IL-6) and tumor necrosis factor (TNF)] have been found to be expressed during the DO process, especially during the latency phase (46). The expression of bone resorption factors was investigated in the rabbit model of DO and showed diminished expression during the consolidation phase of DO (47, 48).

The expression of mechanotransduction factors during the distraction process has also been reported and includes extracellular signal-regulated kinase (ERK), proto-oncogene tyrosine-protein kinase Src (c-Src), integrin pathway, and focal adhesion kinase (FAK) (49–51).

## Effect of the Mechanical Environment in Other Bone Models

Although DO attracted most of the attention in the literature when assessing the effect of the mechanical environment on molecular signaling, there have been numerous studies on these effects in other bone models, including normal and fractured bones. The addition of mechanical loading in these models leads to an alteration of the protein and molecular signaling in the loaded segment, especially during the early loading phases (52–57). In a study by Mantila Roosa et al., they found increased bone matrix genes when loading was applied to the intact forelimb of rats compared to the contralateral unloaded limb (56). In the same study, the loaded limb also showed up-regulation of TGF-β1, PDGF, and bFGF (56). In the same animal model, but in intact hindlimb loading, Raab-Cullen et al. also showed up-regulation of TGF-β1 and IGF when a mechanical load was applied (58). These findings were further proven by multiple *in vivo* and *in vitro* studies where levels of these growth factors were found to be stimulated when loading was applied (53, 59). On the other hand, a downregulation of sclerostin was observed in axially loaded bones thus leading to decreased inhibition of the Wnt/β-catenin signaling pathway and ultimately improving bone quality (55, 57).

In the fractured bones, mechanical loading also showed an agonist effect on bone formation. Palomares et al. studied these effects on protein and molecular signaling during fracture healing in their rat femoral model and found up-regulation of collagen type 2 in the loaded segment of the fracture (60). In his model, BMP 3 was also upregulated by the loading process (60). In other studies, stimulation of BMPs was observed when mechanical loading was applied, but this stimulation was mostly attributed to an indirect effect of the loading process through the Wnt/β-catenin signaling pathway which also showed up-regulation when mechanical stimulation was applied (52–54).

## Methods of Altering the Mechanical Environment

It has been previously shown that alterations of the mechanical environment may have an effect on the healing process of DO (18–20).

Changes in the mechanical environment include addition of compression forces to the distraction forces applied, changes in the technique of distraction (acute or gradual), changes in the rate of distraction (continuous or intermittent), or changes in the rhythm of distraction (for example, accelerated distraction).

### Addition of compression forces

In the context of DO, addition of compression forces to the distraction protocol may take one of several forms, including weight bearing on the distracted limb, alternate cycles of distraction and compression (accordion), over-lengthening, and then shortening or fixator dynamization (18, 19).

#### Weight bearing

Weight bearing during the process of DO has been shown to be an important stimulus for regenerate formation and maturation in DO (1, 6, 61, 62). Radiographical and histological evidence of significantly improved bone formation has been shown when weight bearing was applied in a goat tibial model of DO (63). In a rat model of DO, Radomisli et al. showed that in the weight bearing group BMP2/4, collagen type 1, and osteocalcin expression was more abundant (64). Leung et al. also showed increased expression of TGF-β1 when weight bearing was applied in the lengthened bone thus emphasizing the importance of early weight bearing in DO (63). Although the exact mechanism of increased weight bearing is still not fully understood, the findings in the studies of Radomisli and Leung – specifically, the increased expression of collagen type 1, suggest that weight bearing may have a more significant effect on osteogenesis then chondrogenesis, as it is associated with early collagen type 1 and osteocalcin expression (64).

#### Distraction with addition of compression forces

When examining the impact of adding compression forces to the distraction protocol on molecular signaling the literature is scarce in this aspect (Table 1). In addition, there is no consensus on a standard compression–distraction protocol.

**Table 1:** **Factor expression in distraction and compression forces in rabbit DO model**.

Factor	Outcome	Reference
BMP-4 (mRNA)	Acceleration of expression of BMP-4 when compression applied	Kim et al. (65)^a^
TGF-β1 (mRNA)	Increased and sustained expression of TGF-β1 when compression applied	Kim et al. (65)^a^
Osteonectin (mRNA)	Sustained expression of osteonectin up to 3 weeks post-compression in the accordion group	Kim et al. (65)^a^
VEGF (protein)	Increased expression of VEGF in the compression group	Mori et al. (66)^b^

*DO, distraction osteogenesis; BMP, bone morphogenic protein; VEGF, vascular endothelial growth factor; TGF-β1, transforming growth factor beta*.

*^a^Control group (distraction of 1 mm/day for 8 days) and experimental group (distraction of 1 mm/day for 10 days followed by a 3-day latency period after which they compressed 1 mm/day for 2 days) – rabbit mandibular DO*.

*^b^Control group (distraction of 0.7 mm/day for 14 days) and experimental group (distraction of 0.7 mm/day for 14 days then compression of 0.7 mm/day for 3 days) – rabbit tibial DO*.

**Figure 7:**
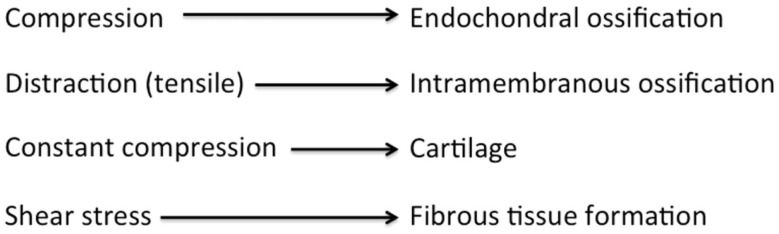
**Diagram showing the process of osteogenesis by different mechanical environments**. Adopted from Chao and Inoue (75).

Several studies – mostly anecdotal – have shown that the addition of compressive forces alternating with the standard distraction protocol – known as the accordion technique – stimulates bone formation in the distracted gap (67–70). The accordion technique has also been successful after bone transport in obtaining union at the docking site and thus managing the bone defect (71, 72). It is believed that compression forces favor intramembranous bone formation, whereas distraction (tensile) forces favor endochondral bone formation (Figure 7) (38, 73–75). Hence, the combination of these two different types of mechanical loading may stimulate bone formation more than either type of loading alone. The literature lacks in studies examining the molecular effect of the accordion maneuver in DO, as most of the studies apply compression at the end of the distraction process and not in an alternating pattern.

Another method of adding compressive forces is the protocol of over distracting the bone by a few millimeters, beyond the planned amount of lengthening followed by gradual shortening (or compression) equivalent to the amount of over-lengthening. One well-designed study by Kim et al. examined the effect of the addition of compressive forces (distraction followed by compression) on BMP-4 in the mandible DO rabbit model (65). They divided their experiment into a control group (distraction of 1 mm/day for 8 days) and an experimental group (distraction of 1 mm/day for 10 days followed by a 3-day latency period after which they compressed 1 mm/day for 2 days). The level of BMP-4 expression increased in both groups at the end of the distraction process, but in the experimental group the expression accelerated when compression was applied and was maintained for 2 weeks post-compression. In the same study, it was reported that the expression of TGF-β1 not only markedly increased when compression was applied but also continued until 2 weeks after compression, whereas in the control group, there was only moderate elevation and remained elevated for a significantly shorter period of the DO process (65). Hamanishi et al. also applied this method in a rabbit model of tibia DO and found histologically increased proliferation of osteoblasts in the distraction gap (76). Surprisingly, this was associated with decreased vascularity, which they attributed to the compression forces causing vascular lumen collapse (76). Also, in a rabbit DO model, Mori et al. demonstrated that although there was collapse of the vascular lumen when compression was applied, VEGF and hypoxia inducible factor-1a (HIF-1a) showed marked increase in expression (66). They concluded that this method enhanced membranous bone formation in the distraction gap.

### Acute versus gradual distraction

Compared to gradual distraction (GD), acute distraction (AD) is a less favorable method for bone regeneration in DO (Table 2) (25, 51, 77). At the molecular level, studies comparing GD and AD in the context of DO are limited. Warren et al. found a low expression of collagen type 1 and osteocalcin in the mandibular DO rat model during the consolidation phase of the AD group compared with the control (25). Fang et al. compared FGF expression in GD with AD and found that expression of FGF increased in GD more than AD (77).

**Table 2:** **Factor expression in acute versus gradual distraction osteogenesis in rat mandibles**.

Factor	Outcome	Reference
ERK 1/2, BMP 2/4 (protein)	GD → ↑ ERK1/2+ ↑ BMP2/4	Rhee et al. (51)^a^
	AD → no ERK1/2	
VEGF/FGF (protein)	GD → ↑ VEGF and FGF	Fang et al. (77)^b^
	AD → absence of GF	
Osteocalcin, collagen type 1, TIMP-1, VEGF (mRNA)	GD → ↑ osteocalcin Collagen type 1 and TIMP-1 compared to AD Both protocols had no effect on VEGF	Warren et al. (25)^c^

*DO, distraction osteogenesis; GD, gradual distraction; AD, acute distraction; ERK1/2 extracellular signal-related kinase; BMP, bone morphogenic protein; VEGF, vascular endothelial growth factor; FGF, fibroblast growth factor; GF, growth factors; TIMP-1, tissue inhibitor metalloproteinase’s-1*.

*^a^Gradual distraction group (distraction of 0.6 mm/day for 8.5 days), Acute distraction groups (intra-operative separation of 2.1 mm in group 1 and 5.1 mm in group 2)*.

*^b^Gradual distraction group (distraction of 0.5 mm/day for 8 days) and acute distraction group (intra-operative separation of 4 mm)*.

*^c^Gradual distraction group (distraction of 0.5 mm/day for 6 days) and acute distraction group (intra-operative separation of 3 mm)*.

### Continuous versus intermittent distraction

Several experimental studies have shown improved bone regeneration using continuous versus intermittent DO (Table 3) (78–80). Continuous distraction leads to up-regulation of several genes more than intermittent traction (78, 79, 81). In a mandibular DO rabbit model comparing continuous versus intermittent distraction, Zheng et al. found that VEGF was significantly upregulated at the early stage of distraction phase and remained elevated throughout the consolidation phase, while FGF was significantly higher at the early stage of distraction phase only and no significant difference was established between the two groups in the other stages. Also, there was advanced bone formation and partial bone healing in continuous DO based on histological examination (78). In the same model, Zheng et al. also reported that, compared to intermittent DO, the mRNA level for BMP-2 in rabbits undergoing continuous distraction, was significantly higher throughout the distraction phase, whereas no significant difference was noted in the consolidation phase (79). Furthermore, the mRNA expression of TGF-β1 was significantly higher at the early stage of distraction phase in the continuous distraction group, although no significant difference was found between two groups in other stages (79).

**Table 3:** **Factor expression in continuous versus intermittent distraction osteogenesis in rabbit mandibles**.

Factor	Outcome	Reference
TIMP-1 (mRNA)	Up-regulating TIMP-1 in continuous DO	Liu et al. (80, 81)^a^
TGF-β1/BMP 2 (mRNA)	High level of TGF-β1 and BMP 2 in continuous DO	Zheng et al. (78, 79)^a^
VEGF/bFGF (mRNA)	Continuous DO → proper mechanical environment for angiogenesis through up-regulation of the angiogenic mediators	Zheng et al. (78, 79)^a^

*DO, distraction osteogenesis; TIMP-1, tissue inhibitor metalloproteinase’s-1; BMP, bone morphogenic protein; TGF-β1, transforming growth factor beta; bFGF, basic fibroblast growth factor; VEGF, vascular endothelial growth factor*.

*^a^Continuous distraction group (0.9 mm/day for 11 days at a rate of 8 times/s) and intermittent distraction group (0.9 mm/day for 11 days at a rate of once per day)*.

Contradicting the previously mentioned positive results of continuous distraction, a recent clinical study by Bright et al. showed no significant difference between intermittent (0.25 mm 4 times/day) and continuous distraction (1/1440 mm 1400 times/day) in time to union or complication rate (82).

### Variable distraction rate and rhythm

The effect of the rate and rhythm used in the applied DO protocol has a significant effect on the expression of factors involved in the DO process (Table 4). Cheung et al. examined the expression of BMP-2, -4, and -7 with routine (0.9 mm/day) and rapid (2.7 mm/day) distraction in the mandible DO rabbit model (83). They showed that in the early consolidation phase there was intense signaling of BMP-2 and BMP-4 at the edges of the distraction regenerate in the routine group, which spread to the primary trabecule as the consolidation phase progressed, whereas in the rapid group there were only weak signals of these BMPs in the area of the distraction regenerate and no extension to other parts of the bone throughout the consolidation phase. In both groups, BMP-7 was not detected throughout the experimental periods.

**Table 4:** **Factor expression in variable distraction rates and rhythms of distraction osteogenesis**.

Factor	Distraction protocol	Model	Outcome	Reference
BMP-2/4/7 (protein)	0.9 mm/day versus 2.7 mm/day	Mandibular DO in rabbits	Increased Expression of BMP-2/4 in 0.9 mm/day group	Cheung et al. (83)
			No BMP-7 in both groups	
FGF/VEGF/PDGF (protein)	0.5 mm/day versus 1.5 mm/day	Femur DO in rats	Increased expression of VEGF, FGF and PDGF in 0.5 mm/day group	Schiller et al. (26)
Endothelial cells antigen (protein)	Four varying rates (0.3, 0.7, 1.3, and 2.7 mm/day)	Tibia DO in rabbits	The vascularization process was maximally stimulated at distraction rates of 0.7 and 1.3 mm/day. While impaired in 0.3 mm/day and not maximally stimulated in 2.7 mm/day	Li et al. (24, 38)
Collagen type 4 (protein)	Four varying rates (0.3, 0.7, 1.3, and 2.7 mm/day)	Tibia DO in rabbits	Collagen type 4 expression was highest at rates of 0.7 mm/day and 1.3 mm/day	Li et al. (24, 38)

*DO, distraction osteogenesis; BMP, bone morphogenic protein; VEGF, vascular endothelial growth factor; FGF, fibroblast growth factor; PDGF, platelet-derived growth factor*.

Schiller et al. studied the alteration of expression of various growth factors in rapid distraction (0.75 mm twice/day) compared to routine (0.25 mm twice/day). They found that there was decreased cellular staining of FGF, VEGF, and PDGF in the rapid distraction group starting on the first day of lengthening (26).

## Discussion

The beneficial effects of mechanical loading on bone formation have been known for more than a century, when Wolff developed the concept that bone adapts to its environment (75). However, of all the forms of mechanical loading – compression, tension (or distraction), bending, torsion, and shear – only compression forces were mostly recognized as having an anabolic effect on bone formation. It was Ilizarov, in the 1950s, who was the first one to introduce the concept that distraction (tension) forces could also lead to bone formation, provided these forces are applied in a controlled environment, and that was the key to the success of the technique of DO (1, 6). As previously mentioned, although DO is a very successful technique in generating large amounts of new bone, the long period of time the external fixator has to be kept on until the newly formed bone consolidates, presented a major problem of this technique. The question then arises: how to accelerate newly formed bone in the distracted gap? Numerous techniques have been described to accelerate bone formation, however, manipulation of the mechanical loading environment is probably the most attractive as it is non-invasive, easily applicable, and furthermore adds no cost to the technique. This led some authors to challenge the standard distraction protocol developed by Ilizarov. Several reports in the literature have shown that the addition of compressive forces to the distraction protocol, specifically the accordion technique, could be beneficial in accelerating bone formation in the distracted gap (67–70, 84). However, there has been no detailed analysis of this technique: how much compression should be applied, which phase of DO should the compression be added and for how long? More importantly, it still remains largely unknown how the effects of compression forces differ from those of distraction forces at the molecular level in stimulating bone formation in the context of DO.

The expression of multiple growth factors has been identified in context of standard DO protocol, including TGF-β, PDGF, IGF, bFGF, and VEGF. While alteration of the mechanical environment lead to variable changes in expression of these factors. Except for the BMP pathway, we were unable to identify any other specific protein, molecule or pathway that clearly characterizes specific changes in signaling when the biomechanical loading environment is altered. The BMP pathway has been extensively studied in the context of bone regeneration and DO and we and others have shown that it plays a significant role in DO using the standard protocol (28, 32, 36, 85). We have also shown (Tables 1–4) that the expression of BMPs changes when the mechanical loading environment is altered, specifically an increased expression of BMPs when compression forces are added to the standard protocol and when continuous distraction is applied. More research needs to be done in that area to identify which specific combination of biomechanical forces leads to optimal expression of BMPs.

Another possible explanation on the beneficial effects of compression loading on bone formation in DO at the molecular level is related to the expression of sclerostin and the difference in sclerostin inhibition with various types of loading. The emergence of the Wnt pathway as a major player in bone regeneration, along with its alteration when sclerostin is inhibited led to extensive research in that area (86, 87). We know today that sclerostin inhibition is one of the numerous pathways through which mechantransduction may lead to new bone formation (55, 88). In our laboratory, we have demonstrated in a mouse model of DO that various members of the Wnt signaling pathway are expressed during the distraction process (86) and that systemic application of sclerostin antibodies caused increased bone formation in the distracted gap (87). However, to the best of our knowledge, there has been no study evaluating the effects of altering the mechanical loading environment in the context of DO on the expression of Wnt pathway members and the degree of sclerostin inhibition. The only study we were able to find comparing the effects of compression versus distraction on the expression of sclerostin, was by Robling et al., who showed in an ulnar loading model in the rat that compression loading causes 80% suppression of sclerostin, while distraction loading caused only 20% inhibition of sclerostin (55). This is an extremely important observation as it supports the hypothesis that the addition of compressive loading to the distraction protocol may be beneficial to bone formation by suppressing more sclerostin than distraction forces only. We believe that future research should also focus on analyzing the effects of altering the biomechanical loading environment on the expression of various members of this pathway and hence try to identify the optimal “non-invasive tissue engineering” method to enhance bone formation in DO.

## Conflict of Interest Statement

The authors declare that the research was conducted in the absence of any commercial or financial relationships that could be construed as a potential conflict of interest.
